# Predictive Value of the Systemic Immune Inflammation Index for Adverse Outcomes in Patients With Acute Ischemic Stroke

**DOI:** 10.3389/fneur.2022.836595

**Published:** 2022-03-18

**Authors:** Yun-Xiang Zhou, Wen-Cai Li, Shao-Huai Xia, Ting Xiang, Can Tang, Jia-Li Luo, Ming-Jian Lin, Xue-Wei Xia, Wen-Bo Wang

**Affiliations:** ^1^Department of Neurosurgery, Affiliated Hospital of Guilin Medical University, Guilin, China; ^2^Department of Neurosurgery, Huizhou Municipal Central Hospital, Huizhou, China; ^3^Department of Neurosurgery, Nanxishan Hospital of Guangxi Zhuang Autonomous Region, Guilin, China

**Keywords:** acute ischemic stroke (AIS), inflammation, outcome, nomogram, systemic immune-inflammation index (SII)

## Abstract

**Background and Purpose:**

The systemic immune-inflammation index, a new index based on platelets, neutrophils and lymphocytes, has been shown to be associated with outcomes of patients with venous sinus thrombosis and cancer. However, its application in acute ischemic stroke has rarely been reported. Therefore, we examined the relationship between systemic immune-inflammation index levels at hospital admission and the outcomes of patients 3 months after onset, and plotted a nomogram to predict the probability of adverse outcomes in patients with acute ischemic stroke.

**Methods:**

We retrospectively analyzed a total of 208 patients with acute ischemic stroke who were admitted between January 2020 and December 2020, and recorded the modified Rankin score 3 months later. A modified Rankin score ≥ 3 was defined as an adverse outcome. Age, sex, NIHSS score, SII, hypertension and coronary heart disease were included in the binary logistic regression, and the nomogram was plotted with a regression equation.

**Results:**

Receiver operating characteristic (ROC) curve analysis indicated that the best cutoff value of the systemic immune-inflammation index was 802.8, with a sensitivity of 70.9% and specificity of 58.2% (area under the curve: 0.657, 95% confidence interval: 0.572–0.742). The nomogram had a C index of 0.802. The average error of the calibration curves of the training set and the validation set was 0.021 and 0.034, respectively.

**Conclusion:**

The systemic immune-inflammation index is associated with short-term adverse outcomes in patients with acute ischemic stroke, and the nomograms can predict the risk of adverse outcomes in patients with acute ischemic stroke.

## Introduction

Stroke is the second leading cause of death and disability worldwide (1). Eighty-seven percent of cases are ischemic stroke (2). In China, ~2.4 million new stroke cases occur each year, and the stroke mortality rate has reached 22.3% (3, 4). According to the data from China's Hospital Quality Monitoring System, in 2018, a total of 3,010,204 stoke patients were admitted to 1,853 tertiary hospitals, of whom 2,466,785 had ischemic stroke, accounting for up to 81.9% of stroke cases (4). Therefore, early risk evaluation and severity estimation in stroke patients and identification of risk factors that can be addressed with intervention would improve adverse outcomes in stroke patients (5).

Increasing studies are focusing on the inflammatory response of the brain after stroke, which is a secondary injury mechanism (6) that can indirectly aggravate brain injury (7). In an experimental stroke model, brain injury has been found to be alleviated by inhibition of inflammatory cells (6). Therefore, inflammatory factor-related immunotherapy has been suggested as a potential option to improve the outcomes of stroke patients (8).

Recently, several new leukocyte-based inflammatory indicators, such as the platelet to lymphocyte ratio (PLR), lymphocyte to monocyte ratio (LMR) and systemic immune inflammatory index (SII) have been used as prognostic predictors of certain diseases. A meta-analysis by QIANG et al. has shown that PLR is a predictor of adverse outcomes in patients with non-small cell lung cancer (9). Ha et al. have incorporated LMR into a predictive model for patients with hepatocellular carcinoma and shown that LMR can predict the survival of patients with advanced hepatocellular carcinoma (10). Ren et al. have shown that lower LMR at admission is associated with poor prognosis 3 months later in patients with AIS (3). SII, calculated as platelets × neutrophils/lymphocytes, includes lymphocytes and neutrophils—two immune inflammatory cells—and platelets, and has been reported to accurately predict the outcomes of patients with venous sinus thrombosis (11). Previous studies have suggested that SII is associated with the severity of stroke at admission (12), but this index is not widely applied to predict functional outcomes in AIS. In addition, clinicians cannot easily assess whether patients with AIS will have adverse outcomes at admission. Therefore, in this study, we aimed to discuss the relationship between SII levels at admission and the outcomes of patients with AIS 3 months after onset, and to plot nomograms to predict the probability of adverse outcomes in patients with AIS.

## Methods

### Patient Selection

In this study, patients with AIS admitted to the Affiliated Hospital of Guilin Medical University between January 2020 and December 2020 were retrospectively analyzed. Patients meeting the following criteria were enrolled: (1) admission to the hospital within 24 h of onset, and diagnosis by CT (completed within 24 h) or MRI (completed within 72 h), (2) symptoms and signs of neurological dysfunction, and (3) age over 18 years. Patients who met the following criteria were excluded: (1) history of cerebral infarction and MRS ≥ 2 scores, (2) history of infectious disease in the prior 2 weeks, (3) complications of hematological diseases, (4) use of immune-suppressants or steroid drugs, (5) history of malignant tumor or autoimmune disease, (6) severe liver and kidney dysfunction, and (7) death during hospitalization or the presence of respiratory infections.

This study was approved by the Ethics Committee of the Affiliated Hospital of Guilin Medical University (2021YJSLL-02) and was conducted in accordance with the Declaration of Helsinki. Because this was a retrospective study, the Ethics Committee approved the waiver of signed informed consent, in accordance with national laws and institutional requirements. In this study, the personal identity information of the patients enrolled was anonymized and replaced with a coding system.

### Data Collection

In this study, baseline clinical data were collected for all patients, including age, sex and related risk factors (e.g., coronary heart disease, hypertension, diabetes, smoking and alcohol consumption, atrial fibrillation and hyperlipidemia). Hypertension was defined by a systolic blood pressure ≥140 mmHg and diastolic blood pressure ≥90 mmHg, or a history of hypertension or use of antihypertensive drugs. Diabetes was defined by a fasting blood glucose ≥7.0 mmol/L, random blood glucose ≥11.1 mmol/L, or a previous history of diabetes or use of hypoglycemic drugs. Coronary heart disease was defined by a previous history of coronary heart disease. Atrial fibrillation was defined by a previous history of atrial fibrillation or diagnosis with atrial fibrillation by electrocardiography. A white blood cell count of (4–10) × 109/L was defined as normal; other counts were considered abnormal. A neutrophil count of 2–7 × 109/L was defined as normal; other counts were considered abnormal (13). An absolute lymphocyte count in the range of 0.8–3.5 × 109/L was defined as normal; other counts were considered abnormal. The normal range of platelets was (100–300) × 109/L, and the normal range of the mean corpuscular volume (MCV) was 80–100 Fl. The normal range of mean corpuscular hemoglobin (MCH) was 26–34. LDL < 3.4 mmol/L was considered normal. HDL >1.0 mmol/L was considered normal; other values were considered abnormal. Stroke severity on admission was evaluated according to the National Institutes of Health Stroke Scale (NIHSS). According to previous studies, stroke with an NIHSS score <6 at admission was defined as mild, and stroke with an NIHSS score ≥6 was defined as moderate to severe (14, 15).

Blood samples were collected from all patients for analysis within 24 h. The white blood cell count, neutrophil count, lymphocyte count, monocyte count, platelet count, MCV, MCH, total cholesterol, triglycerides, blood creatinine and blood uric acid were measured. SII, LMR and PLR were calculated on the basis of routine blood examination results at admission. SII = platelets × neutrophils/lymphocytes, LMR = lymphocytes/monocytes and PLR = platelets/lymphocytes.

### Assessment of Stroke Severity and 3-Month Outcome

All patients were scored with the NIHSS scale at admission to assess stroke severity. All enrolled patients were followed up *via* telephone by the same investigator. The modified Rankin scale (mRS) was used to assess the neurological outcomes of the stroke patients after 90 days. mRS score <3 was defined as a good outcome, and mRS ≥3 was defined as an adverse outcome (16).

### Statistical Analysis

SPSS 23.0 (SPSS Inc., Chicago, IL) was used for data analysis, R studio (version 4.1.0) was used for nomogram plotting, and GraphPad Prism 8.3.0 was used to plot the forest map and ROC curves of the three indexes. First, the Kolmogorov-Smirnov test was used to test the normality of the data. Measurement data conforming to a normal distribution are expressed as the mean ± standard deviation, and compared between groups with single factor ANOVA. Measurement data that did not conform to a normal distribution are expressed as median and quartile, and compared between groups with the Kruskal–Wallis test. The measurement data are expressed as percentage *n* (%), the chi-square test was used for comparison between groups, and the Fisher test was used if the expected frequency of more than 20% of the cells was <5. The Bonferroni method was used for multiple comparisons for data found to be statistically significant with the chi-square test. The SII was divided into three groups: group 1 (SII ≤ 575.94), group 2 (SII ≤ 1096.28), and group 3 (SII > 1096.28). ROC curve analysis was used to evaluate the predictive value of SII, LMR and PLR for adverse outcomes in patients with AIS. The cut-off value of SII was determined on the basis of the ROC curve, according to which SII was divided into low SII and high SII groups. Independent predictors of adverse outcomes in stroke were evaluated with a binary logistic regression model, and odds ratios and 95% confidence intervals were calculated. The single-factor analysis results revealed *P* < 0.05 factors and factors related to functional endings, which were included in multi-factor analysis. All statistical results were tested with a two-sided test, and p < 0.05 indicated statistically significant differences.

## Results

### Baseline Characteristics

**Figure 1:**
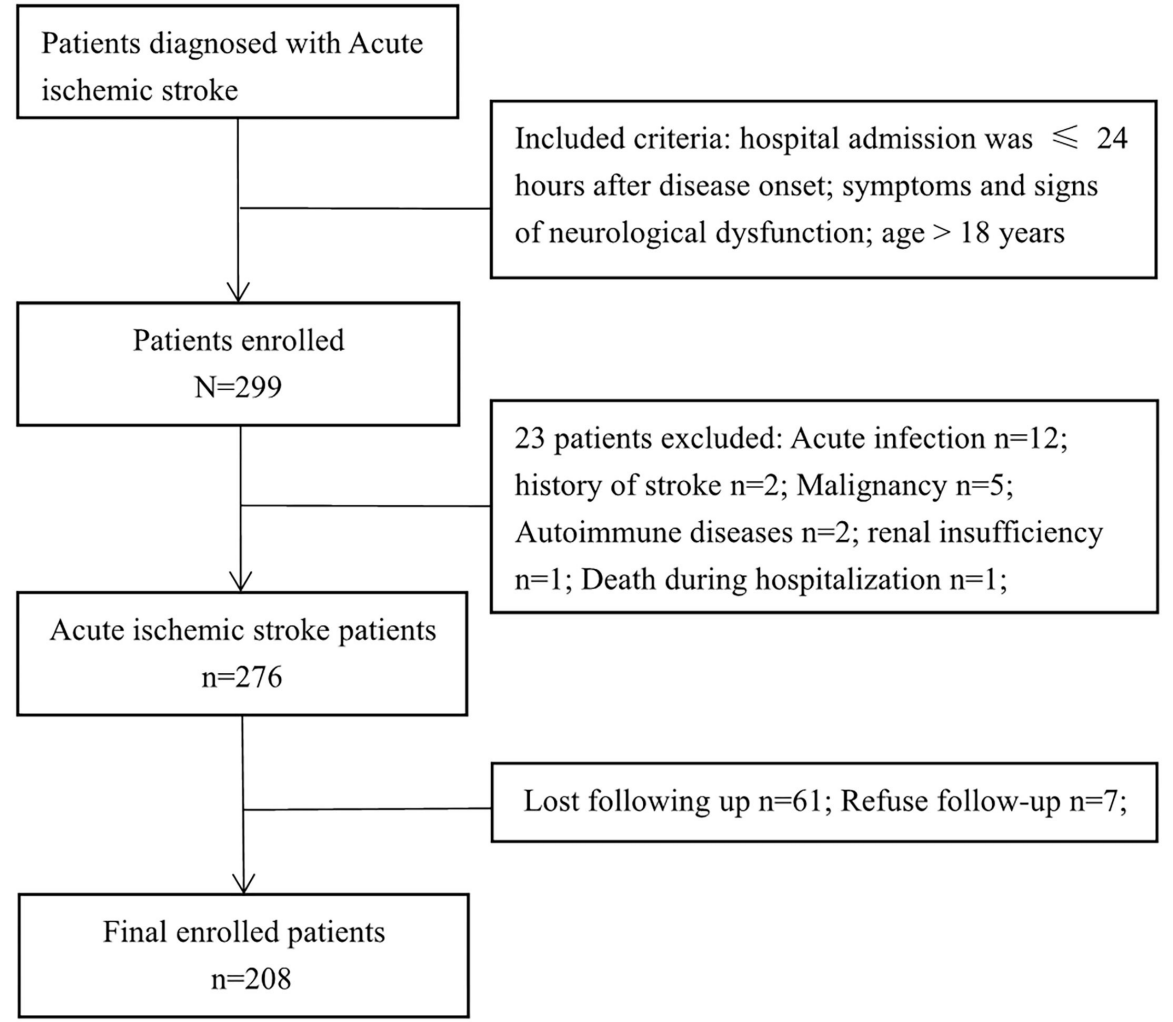
Flow chart for patients' selection.

The clinical data of 299 patients with AIS were collected, 208 of whom were enrolled in the study. Figure 1 shows the exclusion process. Table 1 shows the clinical baseline data of the patients. The mean systolic blood pressure was 150.4 ± 21.2 mmHg, and the mean diastolic blood pressure was 86.9 ± 13.2 mmHg at admission. The average patient age was 63.3 ± 11.3 years, and 68.8% of the patients were male. On the basis of the SII value, the 208 patients with AIS were divided into three groups: group 1 (69 patients with SII ≤ 575.94), group 2 (70 patients with SII ≤ 1096.28), and group 3 (69 patients with SII >1096.28). No significant differences were observed in the clinical baseline data among the three groups. The leukocyte, neutrophil, SII and PLR values of the patients in group 3 were significantly higher than those in the first two groups, whereas the lymphocyte and LMR values of the patients in group 3 were significantly lower than those in the first two groups. There were no significant differences in MCV, MCH, LDL and HDL among the three SII subgroups.

**Table 1:** Baseline clinical characteristics of patients with AIS.

	**Total**	**SII on admission**			***P*-value**
		**SII ≤575.94**	**SII (575.94–1096.28)**	**SII > 1096.28**	
Age (years)	63.3 ± 11.3	62.7 ± 9.9	61.8 ± 11.6	65.4 ± 11.9	0.138
Gender (male)	143 (68.8%)	48 (69.6)	50 (71.4)	45 (65.2)	0.720
Systolic BP (mmHg)	150.4 ± 21.2	148.2 ± 22.0	150.8 ± 19.9	152.3 ± 21.6	0.516
Diastolic BP (mmHg)	86.9 ± 13.2	88.0 ± 13.4	87.6 ± 13.0	85.2 ± 13.3	0.419
Admission to hospital (h)					
Hypertension (*n*)	125 (60.1)	41 (59.4)	43 (61.4)	41 (59.4)	0.962
CHD (*n*)	18 (8.7)	6 (8.7)	5 (7.1)	7 (10.1)	0.820
Diabetes (*n*)	34 (16.3)	8 (11.6)	13 (18.6)	13 (18.8)	0.426
AF (*n*)	15 (7.2)	6 (8.7)	2 (2.9)	7 (10.1)	0.183
NIHSS	2.0 (1.0, 5.0)	2.0 (1.0, 3.5)	2.0 (0.8, 5.0)	3.0 (1.0, 6.0)	0.333
Smoking (*n*)	86 (41.3)	30 (43.5)	32 (45.7)	24 (34.8)	0.386
Alcohol drinking (*n*)	76 (36.5)	25 (36.2)	30 (42.9)	21 (30.4)	0.314
Laboratory tests					
WBC (10^9^/L)	8.0 (6.6, 9.9)	6.9 (5.7, 8.0)	8.0 (6.4, 9.2)	10.0 (8.1, 12.0)	<0.001
Platelet (10^9^/L)	233.0 (201.0, 267.0)	218.0 (189.0, 247.5)	240.0 (207, 275.5)	243.0 (202.5, 283.5.0)	0.005
Neutrophils (10^9^/L)	5.4 (4.0–7.0)	3.8 (3.3, 4.4)	5.5 (4.7, 6.4)	7.6 (6.6, 10.2)	<0.001
Lymphocytes (10^9^/L)	1.6 (1.2, 2.1)	2.1 (1.7, 4.4)	1.6 (1.3, 2.0)	1.1 (0.9, 10.2)	<0.001
SII	797.6 (489.7, 1293.2)	400.5 (277.9, 494.6)	797.6 (711.5, 912.3)	1736.0 (1292.1, 2348.4)	<0.001
LMR	3.0 (2.2, 4.0)	3.9 (3.0, 5.0)	2.9 (2.4, 3.8)	2.0 (1.5, 2.6)	<0.001
PLR	149.1 (112.0, 195.3)	104.2 (78.6, 133.3)	146.0 (125.7, 171.9)	215.3 (180.9, 272.5)	<0.001
MCV	90.3 (87.0, 93.3)	91.2 (88.4, 94.0)	89.8 (86.8, 93.0)	89.0 (86.2, 92.9)	0.098
MCH	30.3 (29.3, 31.8)	31.2 (29.9, 32.2)	30.2 (29.3, 31.7)	30.1 (29.2, 31.3)	0.020
LDL	2.7 (2.2, 3.3)	2.7 (2.3, 3.1)	2.7 (2.1, 3.5)	2.7 (2.0, 3.4)	0.858
HDL	1.0 (0.9, 1.2)	1.0 (0.8, 1.2)	1.0 (0.8, 1.3)	1.1 (0.9, 1.3)	0.303
Etiology					
LAA	15 (7.2)	2	6	7	
CE	3 (1.4)	0 (0)	1 (1.4)	2 (2.9)	
SAO	15 (17.2)	5 (7.2)	3 (4.3)	7 (10.1)	
SOUE	175 (84.1)	62 (89.9)	60 (85.7)	53 (76.8)	

*AF, atrial fibrillation; CHD, coronary heart disease; MCV, corpuscular volume; MCH, corpuscular hemoglobin; HDL, high-density lipoprotein; LDL, low-density lipoprotein; SII, systemic immune-inflammation index; NIHSS, National Institutes of Health Stroke Scale; LAA, large-artery atherosclerosis; CE: cardio-embolism; SAO, small-artery occlusion; SOUE, stroke of other determined or undetermined etiology*.

*Data are presented as means (±SD) and medians (IQR) or as number (percentage)*.

### Functional Outcomes

**Figure 2:**
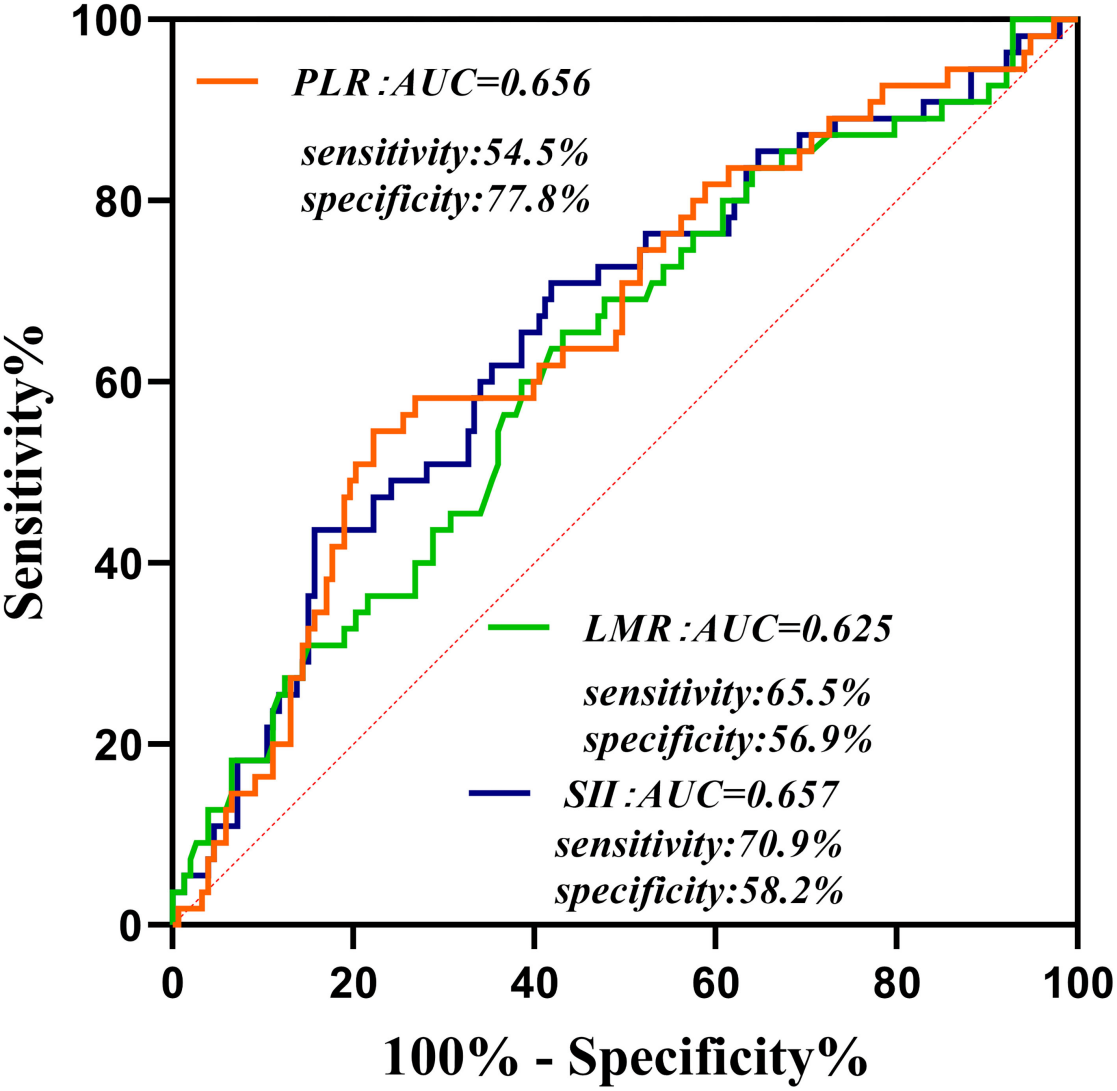
Receiver operating characteristic curve for composite inflammatory ratios (systemic immune inflammatory index, platelet to lymphocyte ratio, and lymphocyte to monocyte ratio) to predict the risk of adverse outcomes in patients with AIS. AUC, area under the curve; SII, systemic immune inflammatory index; PLR, platelet to lymphocyte ratio; LMR, lymphocyte to monocyte ratio.

At the follow-up after 90 days, 55 patients had adverse outcomes: 11 cases in group 1 (15.9), 17 cases in group 2 (24.3%) and 27 cases in group 3 (39.1%). Significant differences were observed in adverse outcomes among the three groups (*p* = 0.007) (Table 2). Group 3 had a higher incidence of adverse outcomes than the first two groups, and significant differences were found between group 1 and group 3 (*p* = 0.002) (Table 3). No significant differences were observed between group 1 and group 2, as well as group 2 and group 3 (*p* = 0.22, 0.06). ROC curve analysis showed that the best cut-off value of SII was 802.8, with a sensitivity of 70.9% and specificity of 58.2% (area under the curve: 0.657, 95% confidence interval: 0.572–0.742) (Figure 2). The best cut-off value of LMR was 2.94, with a sensitivity of 65.5% and specificity of 54.5% (area under curve: 0.625 and 95% confidence interval: 0.289–0.461), and the best cut-off value of PLR was 185.66, with a sensitivity of 54.5% and specificity of 77.8% (area under curve: 0.656 and 95% confidence interval: 0.571–0.74). To further explore the relationship between SII and the clinical status of patients with AIS, we divided 208 patients with AIS into two groups according to their ROC cut-off values (SII > 802.80 × 109/L, *n* = 103 and SII ≤ 802.80 × 109/L, *n* = 105). The median SII values of the two groups were 1294.29 and 504.52, respectively.

**Table 2:** Comparison of outcomes among the three subgroups on the basis of SII.

**Outcomes**	**Total**	**SII on admission**			***P*-value**
		**SII** **≤575.94**	**SII** **(575.94–1096.28)**	**SII > 1096.28**	
mRS ≥ 3 (*n*)	55 (26.4)	11 (15.9)	17 (24.3)	27 (39.1)	0.007

*Data are presented as number (percentage)*.

*mRS, modified Rankin Scale; SII, systemic immune-inflammation index*.

**Table 3:** Comparison of outcomes between low SII and high SII.

**Outcomes**	**Total**	**SII on admission**		***P*-value**
		**SII ≤575.94**	**SII > 1096.28**	
mRS ≥ 3 (*n*)	55 (26.4)	11 (15.9)	27 (39.1)	0.002

*Data are presented as number (percentage)*.

*mRS, modified Rankin Scale; SII, systemic immune-inflammation index*.

**Figure 3:**
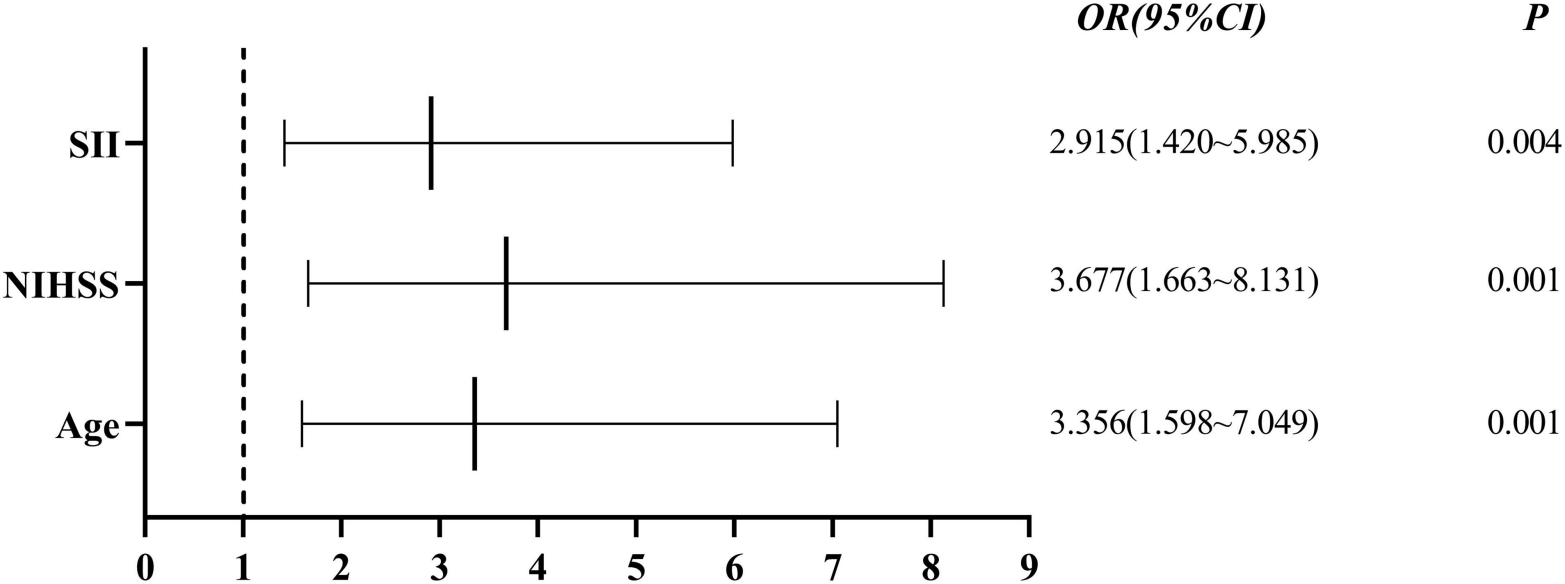
Forest plot for clinical factors. SII, systemic immune inflammatory index; NIHSS: National Institutes of Health Stroke Scale.

Single-factor analysis showed that sex, age above 65 years, high NIHSS scores and SII were associated with outcomes of patients (Table 4). A multi-factor analysis including age, grade, NIHSS score, diabetes, smoking, hypertension and SII showed that high SII was an independent risk factor for 90-day adverse outcomes in stroke patients (odds ratio = 2.915, 95% confidence interval 1.420–5.985 and *p* = 0.004) (Table 5). Figure 3 shows the results of the multi-factor logistic regression analysis.

**Table 4:** Binary logistic regression analysis predicting outcomes.

**Variable**		**Outcomes**		
		**Patients**	**OR (95% CI)**	**Log-rank *P***
Gender				0.05
	Male	143	1	
	Female	65	1.900 (0.999–3.612)	
Age (years)				<0.001
	≤ 65	98	1	
	>65	110	0.280 (0.141–0.557)	
WBC				0.240
	Normal	156	1	
	Abnormal	52	1.508 (0.760–2.992)	
Neutrophils				0.153
	Normal	155	1	
	Abnormal	53	1.640 (0.833–3.231)	
Lymphocytes				0.154
	Normal	188	1	
	Abnormal	20	2.000 (0.771–5.190)	
Platelet				0.269
	Normal	186	1	
	Abnormal	22	1.690 (0.667–4.281)	
SII				<0.001
	Low-SII	105	1	
	High-SII	103	3.390 (1.744–6.589)	
NIHSS				<0.001
	<6	164	1	
	≥6	44	3.970 (1.964–8.022)	
Hypertension				0.532
	No	83	1	
	Yes	125	1.225 (0.648–2.316)	
CHD				0.216
	No	190	1	
	Yes	18	1.883 (0.691–5.130)	
AF				0.228
	No	192	1	
	Yes	15	1.946 (0.659–5.745)	
	Missing	1		
Diabetes				0.096
	No	173	1	
	Yes	34	1.931 (0.890–4.187)	
	Missing	1		
Smoking				0.052
	No	203	1	
	Yes	145	1.553 (0.994–2.427)	
Alcohol drinking				0.183
	No	153	1	
	Yes	55	0.636 (0.327–1.238)	
MCV				0.926
	Normal	186	1	
	Abnormal	22	1.048 (0.388–2.831)	
MCH				0.547
	Normal	186	1	
	Abnormal	22	1.342 (0.516–3.488)	
LDL				0.555
	Normal	161	1	
	Abnormal	47	1.242 (0.605–2.548)	
HDL				0.626
	Normal	119	1	
	Abnormal	89	0.856 (0.457–1.602)	

*AF, atrial fibrillation; CHD, coronary heart disease; MCV, corpuscular volume; MCH, corpuscular hemoglobin; HDL, high-density lipoprotein; LDL, low-density lipoprotein; SII, systemic immune-inflammation index; NIHSS, National Institutes of Health Stroke Scale*.

**Table 5:** Binary logistic regression analysis predicting outcomes.

	**OR**	**95% CI**	***P*-value**
Age	3.356	(1.598–7.049)	0.001
Grade	1.651	(0.719–3.791)	0.237
NIHSS	3.677	(1.663–8.131)	0.001
SII	2.915	(1.420–5.985)	0.004
Diabetes	1.900	(0.794–4.550)	0.150
Smoking	0.772	(0.341–1.751)	0.536
Hypertension	1.365	(0.651–2.861)	0.410

*NIHSS, National Institutes of Health Stroke Scale; SII, systemic immune-inflammation index*.

### Predictive Nomogram Development

**Figure 4:**
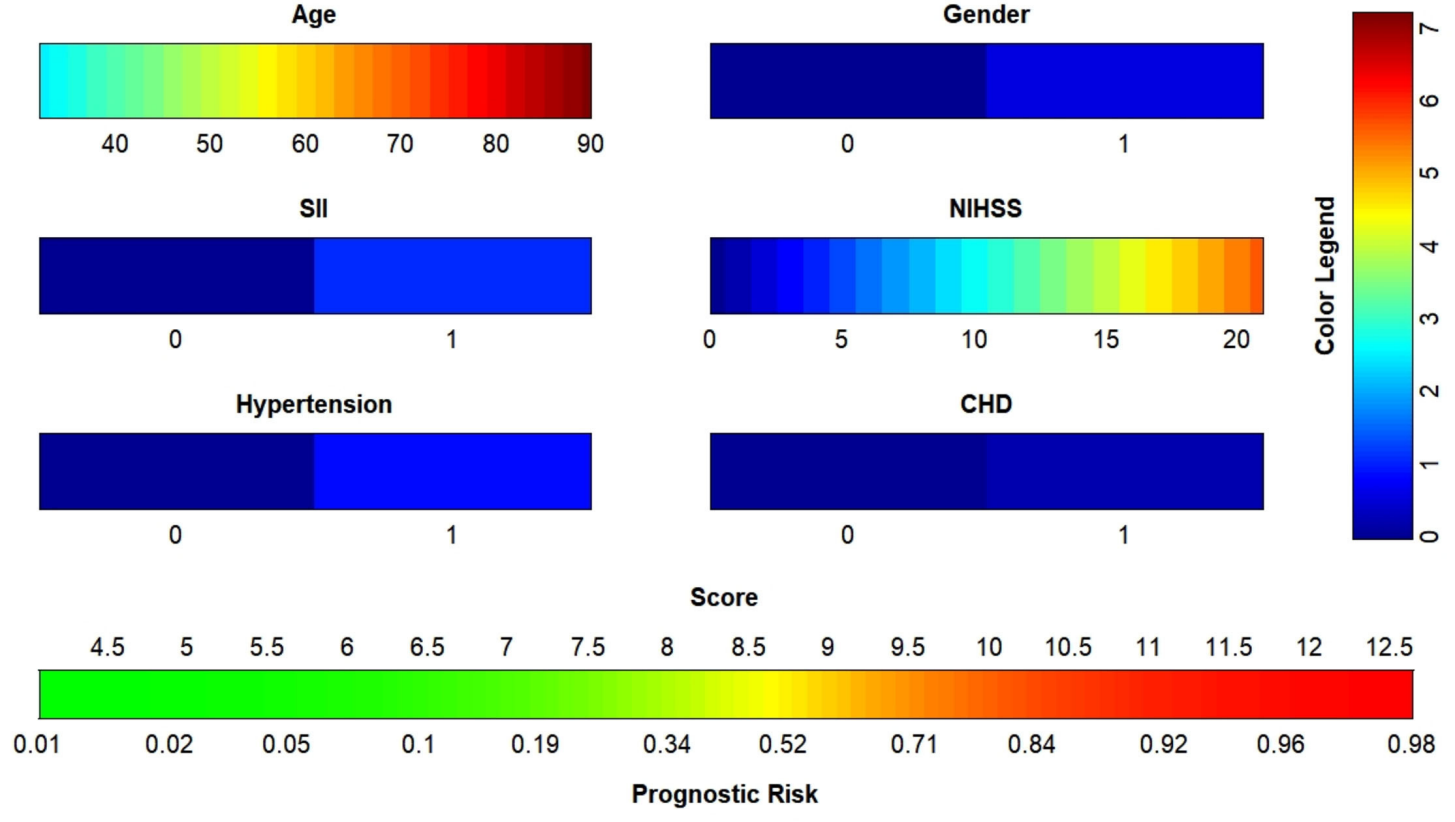
Nomogram to predict the risk of adverse outcome for AIS patients in 3 months. SII, systemic immune inflammatory index; NIHSS, National Institutes of Health Stroke Scale; CHD, coronary heart disease; AIS, acute ischemic stroke.

The data were randomized into training (*n* = 156) and validation (*n* = 52) sets at a ratio of 3: 1 in R (version 4.1.0). Age, sex, NIHSS score, SII, hypertension and coronary heart disease were included in the binary logistic regression, and the nomogram (Figure 4) was plotted with a regression equation in which age and NIHSS score were continuous variables, and SII, sex, hypertension and coronary heart disease were binomial variables. The numbers 0 and 1 at SII in the nomogram indicated low SII and high SII, respectively; 0 and 1 in the sex groups indicated male and female, respectively; and 0 and 1 for hypertension and coronary diseases indicated patients without and with these two diseases, respectively. The nomogram for the training set was plotted, and the C-index was calculated to plot the calibration curve and the ROC curve. Different colors are used for each scoring item in the nomogram; each color indicates a corresponding score in the right score table, and the sum of all scores indicates the corresponding risk at the bottom of the nomogram. The resulting risk was the probability of an adverse outcome in patients with AIS after 90 days.

### Validation of the Nomogram

**Figure 5:**
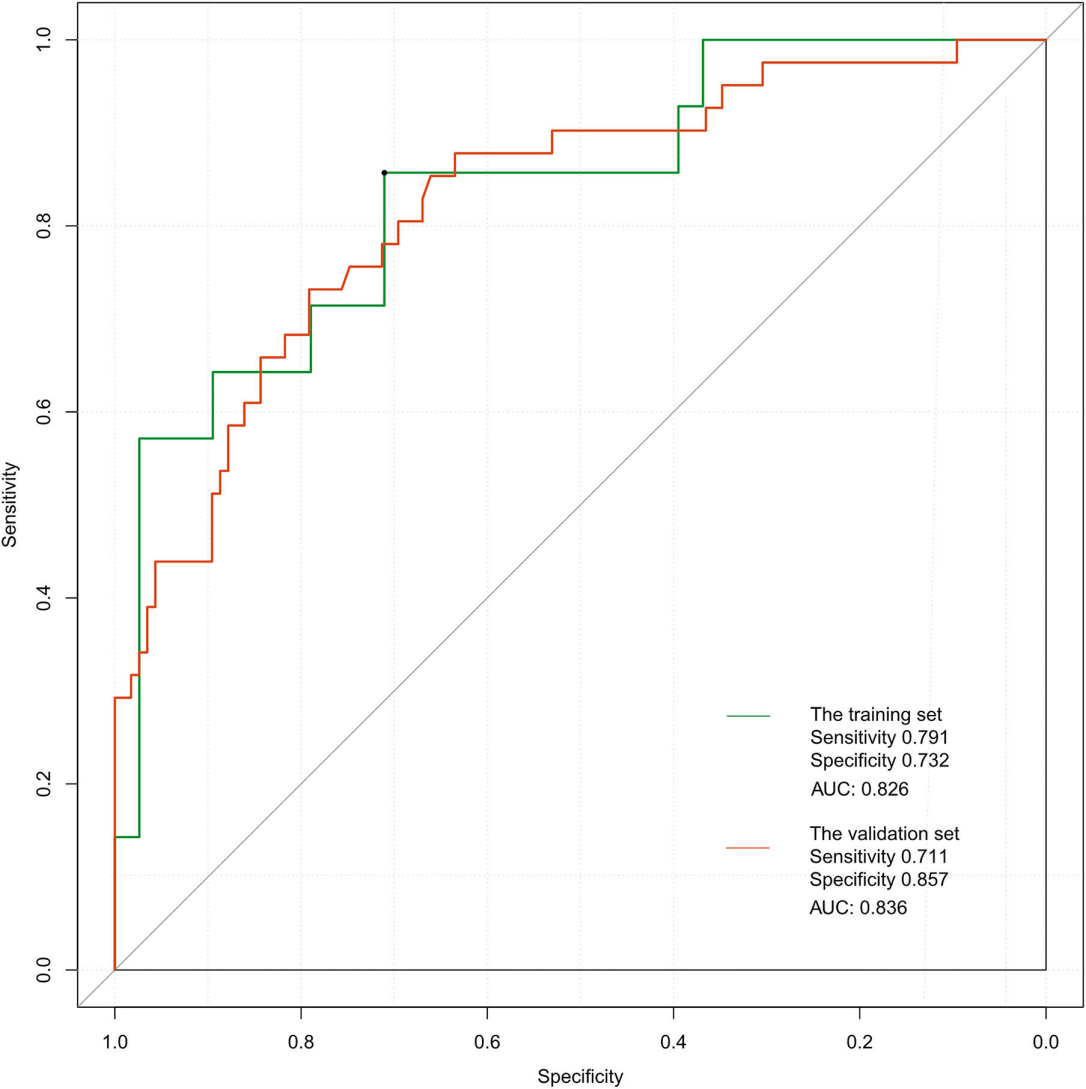
Receiver operating characteristic curves of the proposed nomogram in the training and validation set.

**Figure 6:**
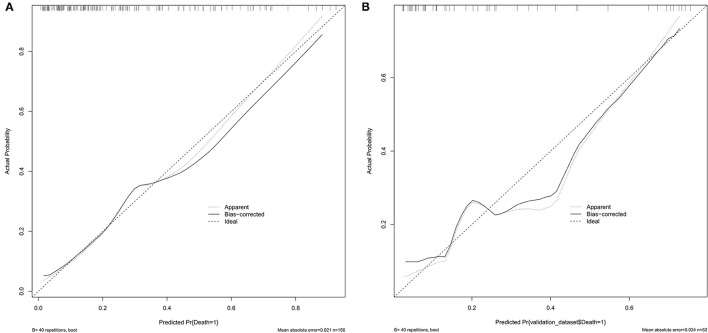
**(A)** Calibration plots of the proposed nomogram in the training set. **(B)** Calibration plots of the proposed nomogram in the validation set.

The ROC curve of the training set (Figure 5) had an AUC = 0.826, and that of the validation set (Figure 5) had an AUC = 0.836. The nomogram had good predictive performance with high discrimination. The nomogram was calibrated with the calibration curve. The calibration curve of the training set (Figure 6A) revealed that the mean error between the risk of adverse outcomes predicted by the predictive model and the actual risk of adverse outcomes was 0.021. Similarly, the calibration curve of the validation set (Figure 6B) showed a mean error of 0.034, indicating consistency between the predicted and observed results. With a C index of 0.802, the nomogram had good accuracy.

## Discussion

In this study, the relationships among SII, LMR and PLR at admission and short and moderate term adverse outcomes in patients with AIS were explored, and their predictive values were compared. SII had a good predictive value for outcomes of patients with AIS, and high SII was closely associated with adverse outcomes in stroke patients. Therefore, SII is considered a new index for outcomes of patients with AIS. Moreover, the nomogram showed good predictive performance.

Previous studies have shown that stroke causes nerve cell death and the release of factors such as damage-related molecular patterns, thereby leading to a local inflammatory response in damaged brain regions (17–19). Mast cells are activated in early stages after the onset of AIS, and subsequently damage the blood-brain barrier and cause brain edema by releasing gelatinase (20, 21). After the destruction of the blood-brain barrier, peripheral leukocytes infiltrate into damaged brain regions and further aggravate the destruction of the blood-brain barrier by periodically releasing pro-inflammatory cytokines, reactive oxygen species and matrix metalloproteinases (22). Neutrophils infiltrate into the ischemic site within several hours after AIS and peak at 24–48 h (23). Increased neutrophil concentrations result in elevated expression of matrix metalloproteinase-9 (24), which further damages the blood brain barrier. In addition, neutrophils further aggravate brain injury by releasing inflammatory mediators (25). After AIS, the infiltration of neutrophils has been associated with injury severity (26). After AIS, blood stagnation flow produces shear stress on endothelial cells and platelets, thus resulting in the deployment of P-selectin of the adhesion molecules to the cell surface (23). Platelets interact directly with circulating leukocytes by changing the surface expression of P-selectin or CD40, thereby forming platelet-leukocyte aggregates and activating the innate immune response to ischemia (27). P-selectin of platelets also acts as a bridge molecule by binding to leukocytes, thus promoting leukocyte aggregation, leading to intravascular occlusion and further contributing to ischemic injury (28). Lymphocytes, a subtype of leukocytes, are also involved in the inflammatory response after stroke. However, the mechanism of action of lymphocytes in AIS in the literature is controversial. Some animal stroke experiments have suggested that increased lymphocytes up-regulate the levels of IL-10 and inhibit the inflammatory cytokines such as IL-6 and TNF-α, thus playing a neuroprotective role (29, 30). Previous studies have shown that lymphocytes aggravate the inflammatory response after stroke (6, 31). The specific effects of lymphocytes on AIS depend on the subtypes of lymphocytes. Furthermore, T cells and γδT CD4+ and CD8+ cells play adverse roles in AIS by producing proinflammatory cytokines such as interferon-gamma and IL-17 (32). In addition, natural killer cells aggregate brain injury by causing neuronal death (33), whereas regulatory T cells (Tregs) play a neuroprotective role by secreting IL-10 (34). Thus, the inflammatory response of AIS is highly complex.

Many studies have shown that inflammatory mechanisms play critical roles in the occurrence and development of ischemic stroke (22, 23, 35, 36). The inflammatory response aggravates ischemic brain damage and neurological dysfunction, whereas chemokines and cytokines released by ischemic tissues promote the infiltration of peripheral circulating leukocytes into ischemic sites (37, 38). The infiltration of leukocytes and the release of various inflammatory mediators cause neuronal death or apoptosis, thus resulting in adverse outcomes in patients with AIS (23). LMR and PLR at admission have been reported to have a good predictive value for the outcomes of patients with AIS. Moreover, low LMR and high PLR are closely associated with the severity of stroke (3, 39). Huang et al. (40) have found that PLR is an independent predictor of depression after a stroke. Yang et al. have found that SII is associated with many adverse events in patients with coronary heart disease (41). Given that stroke is closely associated with inflammation, SII, which combines platelet count, neutrophils and lymphocytes, is a marker of systemic immune inflammation and may be a better predictive index of outcome in AIS.

In summary, exploring the mechanism of inflammatory response after stroke is valuable for applications in immunoregulatory therapy. The SII, which combines neutrophils, lymphocytes and platelets to reflect inflammation and thrombosis, is more reliable and representative than the LMR and PLR. Furthermore, SII has the advantage of easy availability and rapidity, because routine blood analysis is essential for patients admitted to the hospital, and patients are not required to pay extra costs, thus improving compliance.

In our study, SII was an independent predictor of 3-month adverse outcomes in stroke patients. The nomogram showed good predictive performance. SII values can be calculated within several hours after admission, and a nomogram score can be derived and used by clinicians to predict the probability of short-term adverse outcomes in patients with AIS at admission. This tool should be helpful for clinicians by enabling early evaluation of patient outcomes, thereby aiding in the choice of better treatment plans.

This study has some limitations. First, this was a single center study with a small sample size (*n* = 208), which may have resulted in bias and inaccuracy in selection. Second, only SII at admission was calculated, and dynamic monitoring was not performed, thus potentially influencing the correlation between SII and outcomes of patients with AIS. Third, we were unable to analyze other markers of inflammation, such as C-reactive protein, IL-6, fibrinogen and ESR, because of the study's retrospective nature. Fourth, although this study divided samples into two groups—a training set and verification set—through a random grouping method, the verification set was not truly independent, thus potentially increasing the predictive value of the model to some extent. Finally, the study focused on patients admitted within 24 h after the onset of AIS. Therefore, the prognostic model in this study might not be applicable to other types of stroke.

## Conclusion

In summary, our results suggest that SII at admission is a simple new index for predicting the short-tern outcomes of patients with AIS. The nomogram further improves the accuracy of adverse outcome prediction, and thus, may help clinicians determine better treatment plans. Given the limitations of the study, prospective and multicenter studies are needed to further validate the findings.

## Data Availability Statement

The raw data supporting the conclusions of this article will be made available by the authors, without undue reservation.

## Ethics Statement

This study was approved by the Ethics Committee of the Affiliated Hospital of Guilin Medical University (2021YJSLL-02) and was conducted in accordance with the Declaration of Helsinki. The Ethics Committee waived the requirement of written informed consent for participation.

## Author Contributions

Y-XZ, W-CL, and W-BW conceived the review. CT, TX, and Y-XZ conducted data collection. W-CL, S-HX, M-JL, X-WX, J-LL, and CT analyzed and interpreted the data in this study. All authors read and approved the final manuscript.

## Funding

This work was supported by the National Natural Science Foundation of China (No. 81860449) and the Natural Science Foundation of Guangxi (2018GXNSFAA050054).

## Conflict of Interest

The authors declare that the research was conducted in the absence of any commercial or financial relationships that could be construed as a potential conflict of interest.

## Publisher's Note

All claims expressed in this article are solely those of the authors and do not necessarily represent those of their affiliated organizations, or those of the publisher, the editors and the reviewers. Any product that may be evaluated in this article, or claim that may be made by its manufacturer, is not guaranteed or endorsed by the publisher.
